# An Enteroendocrine Cell – Enteric Glia Connection Revealed by 3D Electron Microscopy

**DOI:** 10.1371/journal.pone.0089881

**Published:** 2014-02-26

**Authors:** Diego V. Bohórquez, Leigh A. Samsa, Andrew Roholt, Satish Medicetty, Rashmi Chandra, Rodger A. Liddle

**Affiliations:** 1 Departments of Medicine, Duke University Medical Center, Durham, North Carolina, United States of America; 2 Veterans Affairs Medical Center, Durham, North Carolina, United States of America; 3 Department of Cell Biology and Physiology, University of North Carolina at Chapel Hill, Chapel Hill, North Carolina, United States of America; 4 Renovo Neural Incorporation, Cleveland, Ohio, United States of America; University of Colorado, Boulder, United States of America

## Abstract

The enteroendocrine cell is the cornerstone of gastrointestinal chemosensation. In the intestine and colon, this cell is stimulated by nutrients, tastants that elicit the perception of flavor, and bacterial by-products; and in response, the cell secretes hormones like cholecystokinin and peptide YY – both potent regulators of appetite. The development of transgenic mice with enteroendocrine cells expressing green fluorescent protein has allowed for the elucidation of the apical nutrient sensing mechanisms of the cell. However, the basal secretory aspects of the enteroendocrine cell remain largely unexplored, particularly because a complete account of the enteroendocrine cell ultrastructure does not exist. Today, the fine ultrastructure of a specific cell can be revealed in the third dimension thanks to the invention of serial block face scanning electron microscopy (SBEM). Here, we bridged confocal microscopy with SBEM to identify the enteroendocrine cell of the mouse and study its ultrastructure in the third dimension. The results demonstrated that 73.5% of the peptide-secreting vesicles in the enteroendocrine cell are contained within an axon-like basal process. We called this process a neuropod. This neuropod contains neurofilaments, which are typical structural proteins of axons. Surprisingly, the SBEM data also demonstrated that the enteroendocrine cell neuropod is escorted by enteric glia – the cells that nurture enteric neurons. We extended these structural findings into an *in vitro* intestinal organoid system, in which the addition of glial derived neurotrophic factors enhanced the development of neuropods in enteroendocrine cells. These findings open a new avenue of exploration in gastrointestinal chemosensation by unveiling an unforeseen physical relationship between enteric glia and enteroendocrine cells.

## Introduction

Enteroendocrine cells are essential for normal life [1], [2]. They are sensory cells that coordinate nutrient sensing with metabolic and behavioral functions, like insulin secretion and the regulation of food intake. Such fine coordination is achieved through the secretion of a broad range of neuropeptides, which largely depends on the location of the enteroendocrine cell. For instance, in the stomach, enteroendocrine cells secrete gastrin, somatostatin and ghrelin; whereas, cholecystokinin (CCK), glucagon-like peptide 1 (GLP1) and peptide YY (PYY) are secreted by enteroendocrine cells of the small intestine and colon. In particular, those enteroendocrine cells of the intestine have attracted major attention because the hormones they secrete have been linked to the resolution of obesity and diabetes following gastric bypass [3], [4]. The possibility thus remains that therapeutic treatments for obesity and diabetes could stem from understanding the biology of the intestinal enteroendocrine cell.

Enteroendocrine cells of the small intestine and colon have been traditionally difficult to study. The reason is because, unlike other sensory cells like taste cells, enteroendocrine cells are dispersed and difficult to identify among vast numbers of epithelial cells. This is rapidly changing, however, with the increasing availability of transgenic mice in which the promoters of enteroendocrine cell hormones drive the expression of green fluorescent protein (GFP) [5]–[8]. For instance, transgenic Cck-GFP mice have enabled the discovery of specific molecular receptors that mediate nutrient sensing in enteroendocrine cells, like the case of the G protein-coupled receptor 40 and ILDR1 that mediate stimulation of enteroendocrine cells by fatty acids [9], [10]. The Cck-GFP and Glp1-YFP mouse lines have also helped to demonstrate that intestinal enteroendocrine cells can synthesize about seven hormone peptides, refuting the traditional idea that one enteroendocrine cell can only synthesize one hormone [11], [12]. We recently developed a Pyy-GFP line, and with the help of high-resolution confocal microscopy, unveiled the existence of a prominent basal cytoplasmic process in enteroendocrine cells of the small intestine and colon [7]. Because of its appearance, we called this process a neuropod. This appears to be a conserved feature of other enteroendocrine cells, including those in the stomach [13]; however, with the exception of somatostatin-secreting cells [14], the composition and function of these neuropods in enteroendocrine cells remains largely unknown.

Considering that the base of an enteroendocrine cell in the intestine is no more than 10 µm and the neuropod can reach up to 70 µm, this finding has raised the possibility that signaling and secretion in enteroendocrine cells may be modulated by specific cell-to-cell interactions. Cell-to-cell physical connections such as synapses often span no more than a few hundred nanometers in length and can be fully appreciated only at the ultrastructural level, *in situ* and in the third dimension. This ambitious task was previously limited to serial sectioning transmission electron microscopy, which is a method to reconstruct the ultrastructure of cell fragments largely limited by the demanding manual labor required. The invention of serial block face scanning electron microscopy (SBEM), however, allows rendering in a routinely and automated manner the ultrastructure of cells and tissues in three dimensions [15]. Already popular in the neurosciences, SBEM is helping to reveal specific synaptic connections of neuronal circuits [16], [17].

Likewise, we reasoned that by bridging confocal microscopy with SBEM, a specific enteroendocrine cell could be identified to study its ultrastructure in the third dimension. The results revealed that the enteroendocrine cell’s neuropod has distinctive traits of neuronal axons, including a physical connection with enteric glia. We extended these structural findings into a novel *in vitro* minigut model and discovered that the length and number of enteroendocrine cell neuropods are enhanced in the presence of glial-derived neurotrophic factors.

## Results

### Serial Block Face Scanning Electron Microscopy of a Specific Gut Chemosensory Cell

Because enteroendocrine cells of the intestine (small and large) have been shown to co-synthesize several neuropeptides, including both CCK and PYY, here we used the Cck-GFP and Pyy-GFP mouse lines in a complementary manner to study enteroendocrine cells of the small and large intestine [7].

**Figure 1:**
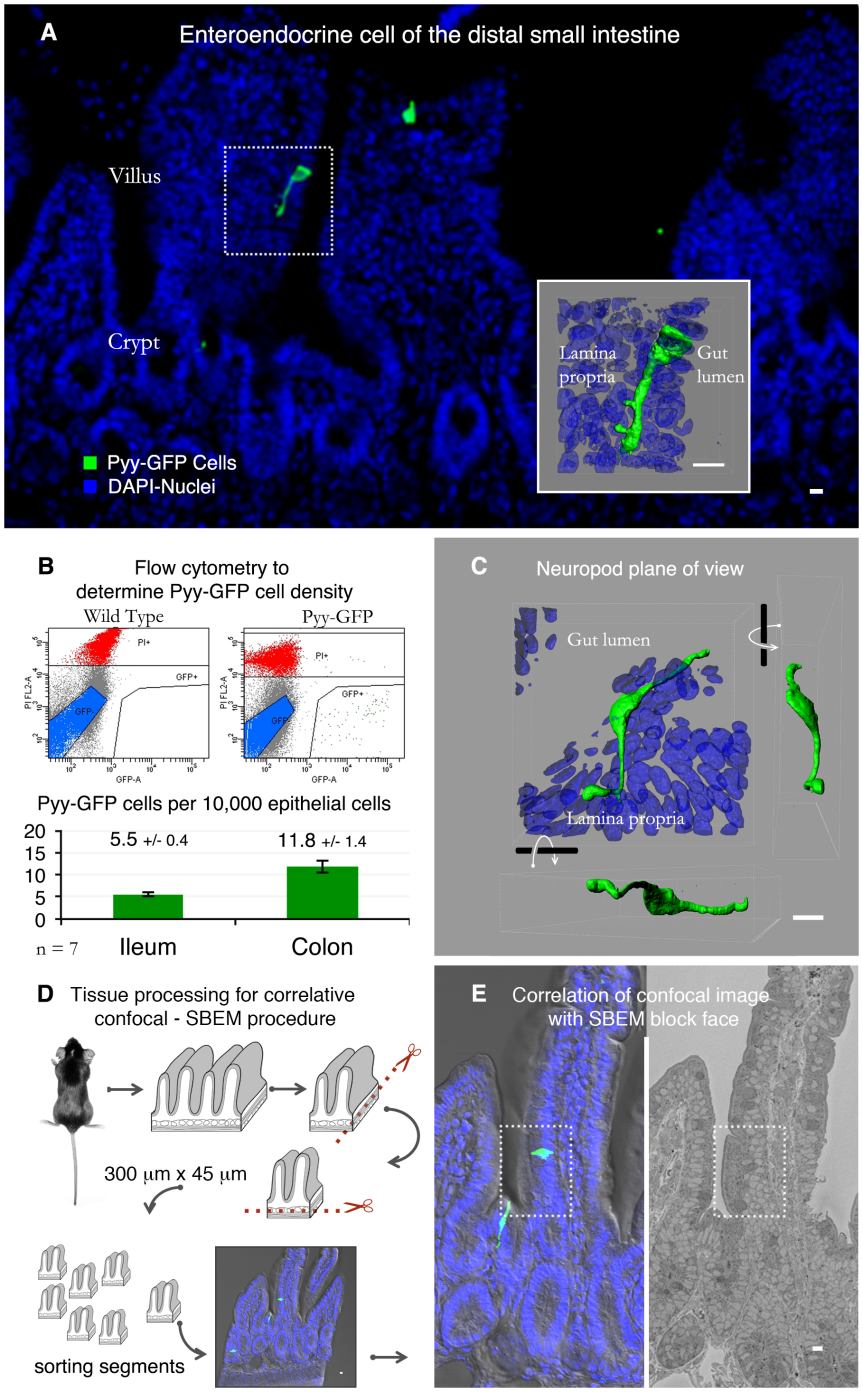
Serial block face scanning electron microscopy of a specific gut chemosensory cell. **A.** Enteroendocrine cell in the ileum of a Pyy-GFP transgenic mouse. Inset shows a reconstruction of a confocal z-stack from the dotted region. **B.** These cells are rare as shown by flow cytometric analysis. For every 10,000 epithelial cells in the colon or ileum, there are only 5.5 or 11.8 Pyy-GFP cells, respectively. Non-viable cells stained with propidium iodide are indicated in red, GFP negative cells in blue, and the area in the lower right corner contains GFP positive cells. **C.** The basal process in Pyy-GFP cells weaves in between epithelial cells, making it difficult to analyze by conventional transmission electron microscopy. **D.** This hurdle can be overcome by serial block face scanning electron microscopy (SBEM). For this, intestinal tissue from a Pyy-GFP mouse was harvested and trimmed with a vibrating blade microtome. The resulting 300 µm wide by 45 µm thick tissue block contained a cell of interest and was imaged with a confocal microscope. E. The block was processed for SBEM. Then, the confocal z-stack was matched to the SBEM image of the entire block face to identify the cell of interest. Bars = 10 µm.

Traditionally, enteroendocrine cells have been identified using immunohistochemistry, and to enhance antibody immunoreactivity tissue sections are cut as thin as 5 µm. Consequently, the cells are often represented as flask-shaped cells (Figure 1A) [18]–[21]. However, the use of transgenic mice expressing GFP in enteroendocrine cells along with confocal microscopy deeper into tissue is revealing the full anatomy of enteroendocrine cells of the distal small intestine (*i.e.* ileum) and colon [22], [23]. Because PYY-secreting enteroendocrine cells are abundant in these portions of the intestine, we used the Pyy-GFP mouse to identify enteroendocrine cells with prominent neuropods and study their ultrastructure. A representative section from the ileum of a Pyy-GFP mouse is shown in Figure 1A. At a glance, it can be appreciated that the density of Pyy-GFP enteroendocrine cells is low. We determined the density of these cells using flow cytometry and found that for every 10 thousand epithelial cells, there are only about 6 to 12 Pyy-GFP ileal or colonic enteroendocrine cells (Figure 1B). The neuropod’s anatomy is elegant but complex; they often reach 70 µm in length and weave beneath 10 to 15 epithelial cells (Figure 1C).

We reasoned that the function of these neuropods could be elucidated from a comprehensive analysis of the cell’s ultrastructure. For this purpose, we optimized a method to bridge confocal microscopy and SBEM. We first harvested intestinal tissue from a Pyy-GFP mouse and used a vibrating blade microtome to obtain tissue segments of 300 µm×45 µm. These dimensions were optimized in pilot studies to facilitate the confocal-SBEM correlation. Tissue segments were sorted and imaged with a confocal microscope to obtain z-stacks before processing them for SBEM (Figure 1D). A low SBEM magnification image of the entire block face was used to measure key features in the data and correlate with the confocal micrographs to find the cell of interest (Figure 1E). The correlation was also facilitated by taking into account structural features, such as position of microvilli, goblet cells, cell nuclei, or basal lamina. The fidelity of this correlative confocal - SBEM method was confirmed in two additional independent experiments, an example of which is presented in Figure S1.

### Reconstructing the Enteroendocrine Cell Ultrastructure in 3D

**Figure 2:**
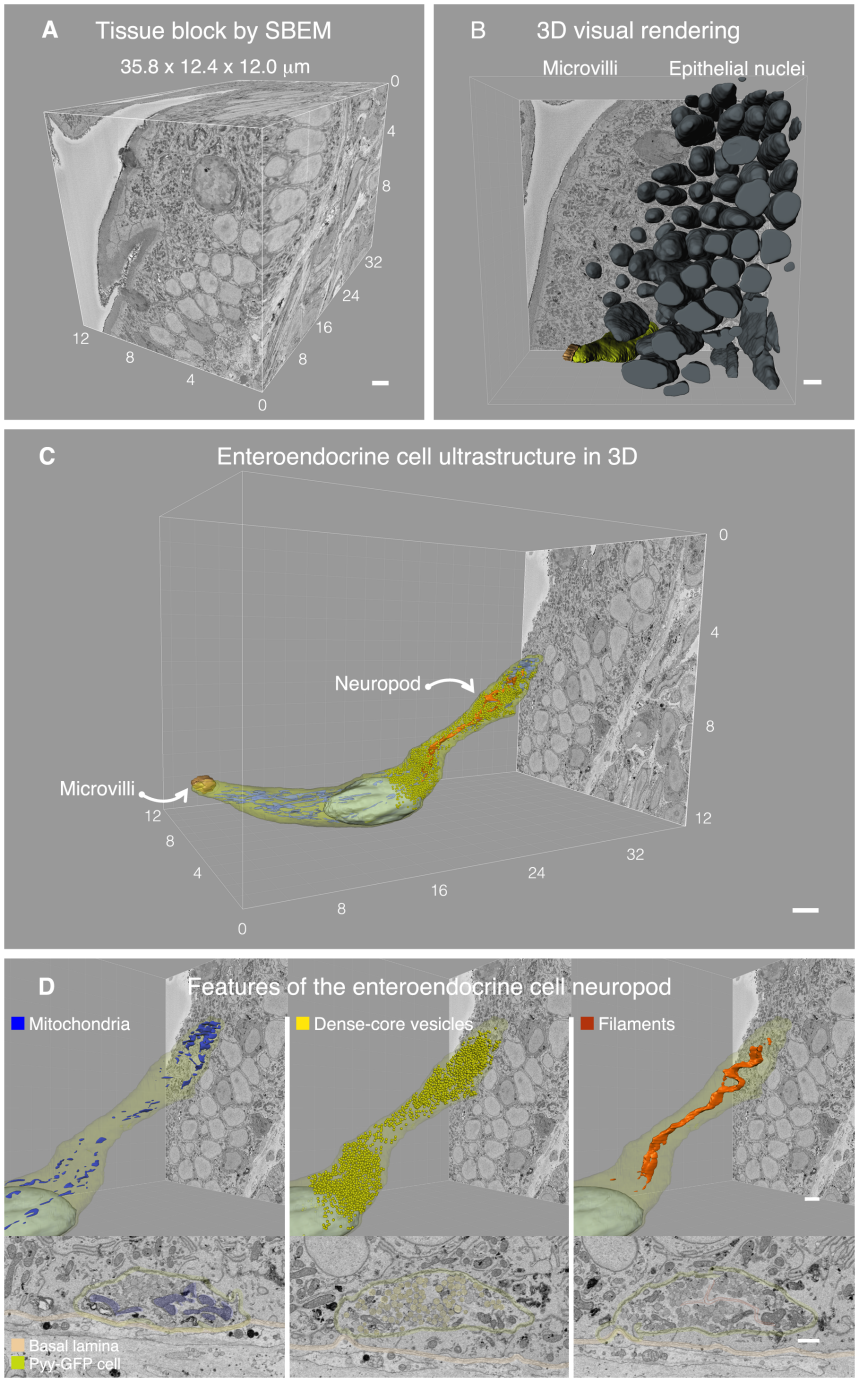
Reconstructing the enteroendocrine cell ultrastructure in 3D. **A.** Data set from serial block face scanning electron microscopy (SBEM) containing a 5 nm/pixel 510 image that spanned a tissue volume of 5,2327 cubic micrometers. **B.** All nuclei were reconstructed in the block using Imaris software. The block contained 145 epithelial cells, including 129 enterocytes, 11 goblet cells, 4 cells of various types, and 1 enteroendocrine cell. **C.** The enteroendocrine cell was traced on each slice to reveal its entire ultrastructure. On the left the cell has a tuft of microvilli exposed to the gut lumen, and on the right there is a prominent neuropod that extends towards the basal lamina propria. **D.** This neuropod is populated by mitochondria, in particular at the tip (blue), secretory vesicles (yellow), and filament-like structures (orange). Top panels show the reconstructions of the cells, and bottom panels show a representative SBEM image of each feature. Structures of interest in the bottom panel have been pseudocolored to facilitate their visualization. Bars = 1 µm.

The cell of interest was contained in a block of tissue of 12×12×36 microns, which was sectioned and imaged every 75 nm, resulting in 700 serial micrographs (Video S1). Imaging was done in an automated fashion, which is a unique advantage of the SBEM technology [16], [17]. The resulting data set had a volume of 5,327 cubic microns with a resolution of 5 nm/pixel (Figure 2A). Microvilli, secretory vesicles, and lipid bilayers were readily distinguishable at this resolution, which facilitated tracing cells and their organelles (Figure S2). We then manually traced and segmented all nuclear membranes in every slice of the data set.

Currently, major efforts are devoted to the development of algorithms that allow automatic segmentation [16], and despite tremendous progress, the existent tools are still limited in their ability to distinguish plasma membranes. Therefore, intensive manual labor is unfortunately still required during segmentation [16], [17]. In this case, about 480 hours of labor were necessary to trace the features of interest. Tracing was performed using Imaris software and, in the process, we found a total of 50 cell nuclei in the lamina propria, classified as follows: 3 enteric glia, 6 subepithelial myofibroblasts, 11 vascular endothelial cells, and 30 other cells of various types. In the epithelium, nuclei were more numerous and we found, including fragments, a total of 145 nuclei. The cell types included 129 enterocytes, 11 goblet cells, 4 cells of various types, and 1 enteroendocrine cell (Figure 2B).

The ultrastructure of the enteroendocrine cell in 3D is presented in Figure 2C and is animated in Video S1. Whereas at the apical end the cell has a noticeable tuft of microvilli exposed to the gut lumen, at its base the cell has a prominent neuropod that extends towards the basal lamina. Mitochondria are distributed throughout the cell but are particularly concentrated at the tip of the neuropod (Figure 2D left). The neuropod also contained the highest concentration of secretory vesicles. There were a total of 8,221 secretory vesicles throughout the cell. The vesicles were distributed as follows: 774 above the nucleus, 1,403 immediately around the nucleus, and 6,044 within the basal process, including 2,492 accumulated at the tip (3 µm from the end). In other words, of all secretory vesicles in the cell, 73.5% are found within the basal process and over 30.3% are accumulated at its tip (Figure 2D center). Thus, the concentration of mitochondria at the tip end of the neuropod may be necessary to sustain the secretion of vesicles.

We also discovered that, within the neuropod, there were thin (<15 nm) filaments that resemble structural proteins (Figure 2D right). They appeared to form bundles of hair-like fibers. These structures originated at the base of the nucleus, extended throughout the neuropod, and appeared to end about 1 µm before its tip end. Because of the axon-like appearance of the neuropod, we reasoned that these filaments may be neurofilaments – the structural proteins found in neuronal axons.

### 3D Ultrastructure Reveals Axonal Process Escorted by Enteric Glia

**Figure 3:**
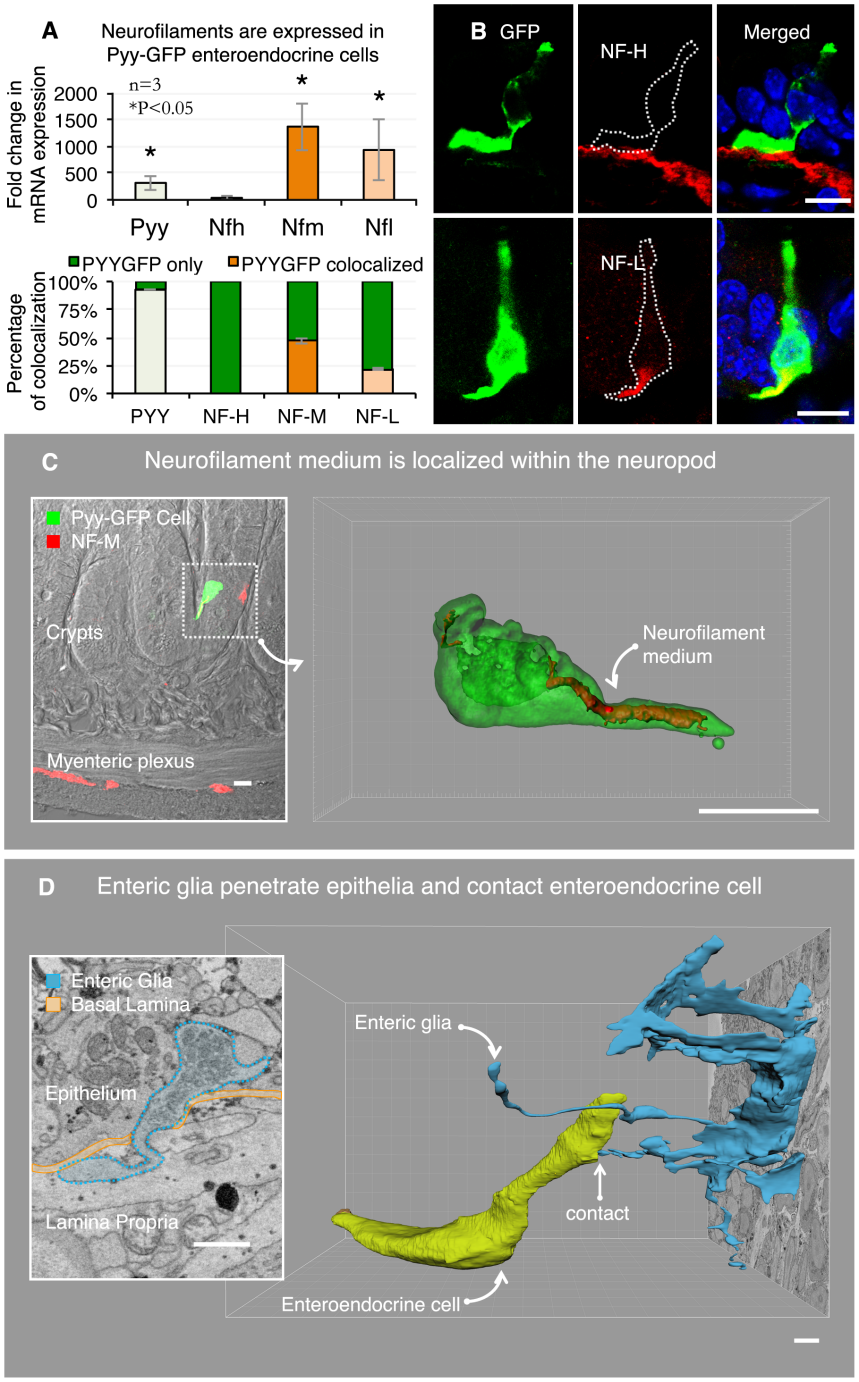
3D ultrastructure reveals axonal process escorted by enteric glia. **A.** Enteroendocrine cells compared to other intestinal epithelial cells express neurofilaments light and medium (top panel). Neurofilament proteins light and medium are expressed in 22% and 47% of Pyy-GFP cells, respectively (bottom panel). This quantification was performed using immunohistochemistry with neurofilament-specific antibodies. **B.** Top panel is a representative image showing that neurofilament heavy is expressed in subepithelial myofibroblasts but not in enteroendocrine cells. Neurofilament light is contained within the Pyy-GFP cell basal process (bottom panel). **C.** Enteroendocrine cells contain neurofilament medium within the neuropod. Inset shows the position of the cell in the epithelium of the ileum. 3D reconstruction of confocal z-stacks depicts the neurofilament medium contained within the Pyy-GFP cell neuropod. **D.** The SBEM data also revealed the relationship between the neuropod in the Pyy-GFP cell and enteric glia. Enteric glia trespass the basal lamina and penetrate into the epithelium (inset). SBEM data segmentation revealed that the enteric glia extends a cytoplasmic process into the epithelium that contacts the enteroendocrine cell neuropod. Bars in B and C = 10 µm, in D = 1 µm.

Neurofilaments are a family of intermediate filaments formed by the following three members: NF heavy (200–220 kDa), NF medium (145–160 kDa), and NF light (68–70 kDa) [24]. Using fluorescence activated cell (FAC) sorting and qRT-PCR, we tested the expression of neurofilaments genes in Pyy-GFP enteroendocrine cells compared to non-GFP epithelial cells. RNA from dissociated sensory neurons served as positive control. Although no expression of NF heavy was observed in enteroendocrine cells, there was significant expression of NF medium and light in Pyy-GFP enteroendocrine cells compared to non-GFP epithelial cells (Figure 3A).

We confirmed these results using immunohistochemistry with neurofilament-specific antibodies and found that for every 100 Pyy-GFP enteroendocrine cells, 22 contained NF light and 47 contained NF medium (Figure 3A). Similar results were found using tissue from Cck-GFP mice (data not shown). It is important to mention that while Pyy-GFP cells did not express NF heavy, this neurofilament type was expressed in subepithelial myofibroblasts (Figure 3A–B). This result was confirmed using an antibody against alpha smooth muscle actin, which is an established marker of subepithelial myofibroblasts [25]. Both NF light and NF medium were found within the basal process of enteroendocrine cells. An example of NF medium found in the enteroendocrine cell is shown in Figure 3C (inset). We acquired optical slices (z-stack) of the dotted region using confocal microscopy and reconstructed it using Imaris software. The resulting 3D rendering showed that NF medium was contained within the neuropod (Video S2).

We noticed also that within the lamina propria there were enteric glial cells. These were classified as enteric glia based on three previously reported characteristics: 1) their characteristic long and thin processes extending radially from the cell body and over neuron fibers [26], 2) their typical elongated nuclei within a planar cell body [27], and 3) the distinctive sheath that these processes form around bundles of 12 to 15 nerve fibers [26]–[28]. This is in contrast with glia in the central nervous system where one glial process forms a sheath around one axon. Examples of these three features are shown in Figure S3.

Segmentation revealed that four glial processes penetrated from the lamina propria into the epithelium (Figure 3D-inset). The entry point into the epithelium was a fenestration in the basal lamina formed by subepithelial myofibroblasts [29]. The glial processes contained visible, small, clear secretory vesicles, similar to those found in synaptic terminals (Figure 3D-inset). Three of the four glial processes did not appear to reach a specific cell type; however, one of them directly reached the enteroendocrine cell neuropod (Figure 3D). Because SBEM is currently not compatible with immunogold techniques, we confirm these findings by means of immunohistochemistry on transgenic Pyy-GFP intestinal tissue, as shown below.

The glial process was slender and less than 50 nm in diameter. Because slices in the data set are spaced every 75 nm, the glial process could only be traced in the z-axis and was fragmented within 500 nm from the enteroendocrine cell. No evident structural specialization was observed at the point of contact; however, it was notable that immediately above the entry point, the glial process bent towards the enteroendocrine cell until reaching its basal process (Video S3). In an additional SBEM data set, we identified a putative enteroendocrine cell also contacted by a glial process (Figure S4). Although in this particular data set the glial process could not be traced back to the glial cell body, it further supports a conserved relationship between enteroendocrine cells and enteric glia. Although their function is only beginning to be explored, enteric glial cells like glia in the central nervous system are needed for the maintenance of enteric neurons [30], [31]. Thus, we wondered if this physical relationship between enteric glia and enteroendocrine cells would be related to the formation of neuropods.

### Bridging Structure to Function: Neurotrophins and the Formation of Neuropods

**Figure 4:**
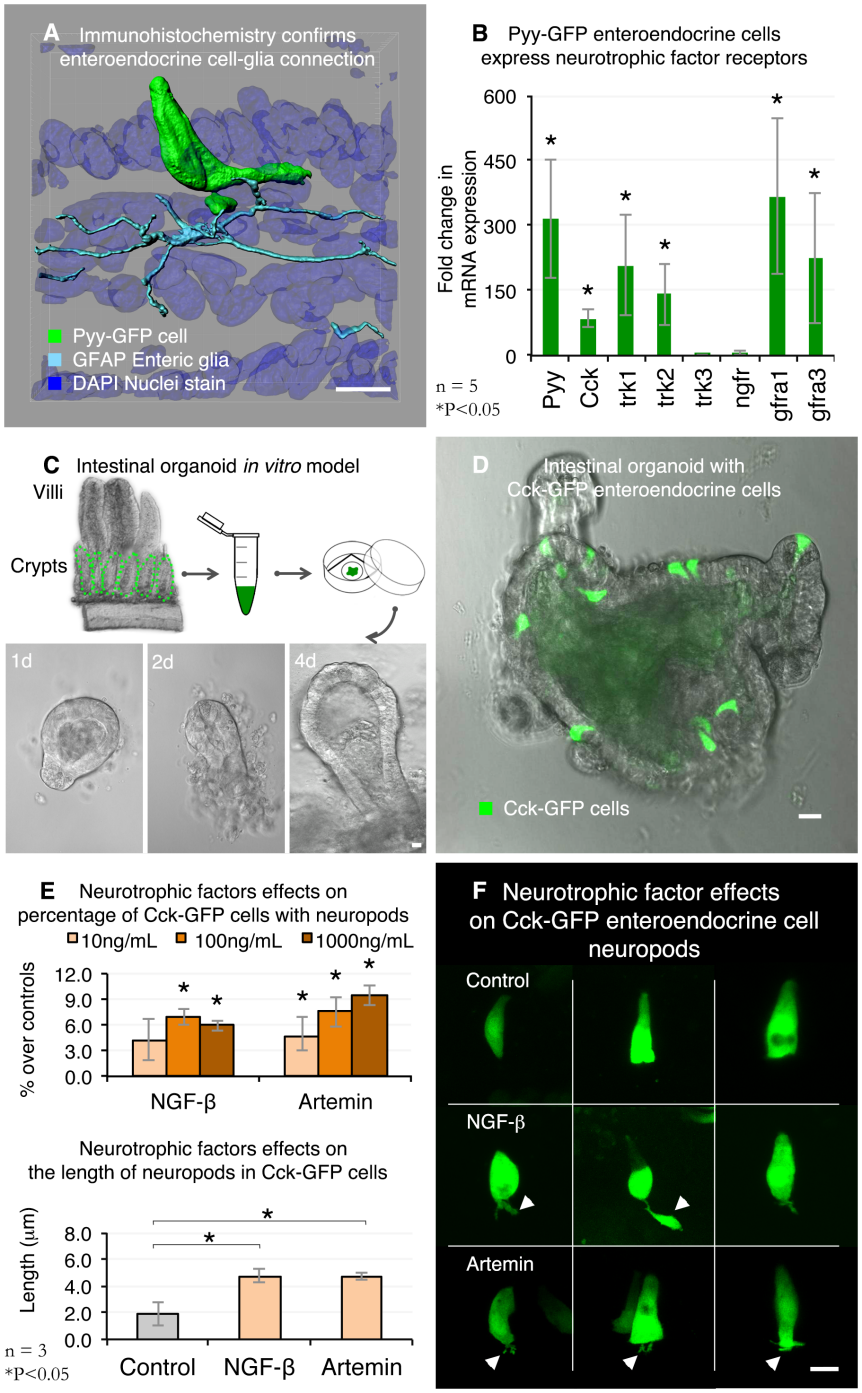
Bridging structure to function: neurotrophic factors and the formation of neuropods. **A.** 3D reconstruction of a confocal z-stack shows glial fibrillary acidic protein (GFAP) immunoreactive enteric glia contacting basal processes in Pyy-GFP cells. Figure S4 contains fluorescence data. **B.** Pyy-GFP enteroendocrine cells express neurotrophic factor receptors trkA, trkB, gfra1, and gfra3. Fold expression is relative to non-GFP intestinal epithelial cells. **C.** Intestinal organoids were used as a model to test the effects of neurotrophic factors on enteroendocrine cells. Treatments were applied to organoids after 4 days of culture. **D.** Organoids were cultured from an analogous Cck-GFP mouse model. Cck-GFP organoids had about six GFP positive cells per organoid compared to less than one for every 10 organoids in Pyy-GFP organoids. Representative image of a 4-day old Cck-GFP intestinal organoid. **E.** Top: Upon the addition of nerve growth factor - β (NGF-β) or artemin, the percentage of enteroendocrine cells with neuropods increased in a dose-dependent manner. Bottom: compared to controls, 10 ng/mL of NGF-β or artemin significantly increased the length of neuropods in enteroendocrine cells. **F.** Representative images of NGF-β or artemin effects after 24 h of a 10 ng/mL treatment. Bars = 10 µm.

Enteric glia are essential for the normal development of enteric neurons and for maintaining the integrity of the intestinal epithelium [32]. They are commonly identified in the small intestine and colon with antibodies against the proteins S100ß and glial fibrillary acidic protein (GFAP) [33], [34]. GFAP is preferred because, unlike S100ß, it does not cross-react with subepithelial myofibroblasts. We used both antibodies to confirm the physical relationship between enteroendocrine cells and enteric glia. As expected, GFAP-positive enteric glia contacted the neuropods in Pyy-GFP enteroendocrine cells. S100ß yielded similar results. Figure 4A highlights a Pyy-GFP cell with two neuropods contacted by individual GFAP glial processes. The fluorescence data are presented in .

Glia and neurons nurture axonal and dendritic development through the secretion of neurotrophic factors that act on specific cell surface receptors. A classic example is that of nerve growth factor, which binds to p75 – a low affinity nerve growth factor receptor or TrkA – the high affinity nerve growth factor receptor [35]–[37]. Thus, we tested the possibility that enteroendocrine cells may express specific receptors for neurotrophic factors by using qRT-PCR and TaqMan probe-based gene expression analysis on enteroendocrine cells. Total RNA isolated from FAC sorted Pyy-GFP and non-GFP intestinal epithelial cells served as controls. The results showed that enteroendocrine cells significantly expressed at least four of the six receptors tested. TrkA and the glial-derived neurotrophic factor 3 (Gfra3) are two examples [36], [37]. We observed comparable results using FAC sorted Cck-GFP enteroendocrine cells (data not shown). Because TrkA and Gfra3 are the receptors for NGF-β and artemin – two established neurotrophic factors known to stimulate axonal elongation and branching in neurons [38]–[40], we tested the possibility that NGF-β or artemin may also induce the formation of neuropods in enteroendocrine cells.

An ideal system to test this hypothesis would be one in which enteroendocrine cells preserve an apical-basal arrangement, such as that of normal gut epithelium. With this in mind, we adapted an *in vitro* organoid system often referred as “mini-guts”. What makes mini-guts an ideal model to study enteroendocrine cells is that cells preserved their polarity within a layer of epithelial cells, yet they lack the input of other cells from the lamina propria that may confound treatment effects, like enteric glia. For this purpose, we used our transgenic Cck-GFP mouse because, after four days in culture, they contained on average more than six GFP positive enteroendocrine cells compared to about one GFP cell for every ten Pyy-GFP mini-guts. An outline of the mini-guts model is presented in Figure 4C. In the mini-guts, enteroendocrine cells had a classic flask shaped anatomy and, just like enteroendocrine cells in tissue, they expressed neurofilaments medium and light (data not shown). However, whereas, in tissue, Cck-GFP or Pyy-GFP enteroendocrine cells have visible neuropods [7], [23], in mini-guts, enteroendocrine cells had either an occasional neuropod or completely lacked basal neuropods (Figure 4D).

We then used mini-guts to test the effects of neurotrophic factors on enteroendocrine cells. It is important to highlight that the concentrations of NGF-β and artemin added to mini-guts’ medium (10–1000 ng/mL) were based on assays established to induce effects in neurons [41], [42]. Compared to controls that were not treated with a neurotrophic factor, the addition of NGF-β for 24 hours significantly increased the percentage of cells that had one or more neuropods. These effects were significantly evident at concentrations equal to or higher than 100 ng/mL. Likewise, upon adding artemin in concentrations as low as 10 ng/mL there was a significant increase in the percentage of enteroendocrine cells with one or more neuropods (Figure 4E). Compared to controls, both NGF-β or artemin added at 10 ng/mL enhanced significantly the length of neuropods (Figure 4E). The length of neuropods did not differ between the NGF-β and artemin treatments but its anatomy was different. We observed that NGF-β induced the formation of one main neuropod with an occasional branch, whereas the artemin treatment resulted in enteroendocrine cells with branched neuropods of similar length (Figure 4F).

It is possible that other neurotrophic factors like glial-derived neurotrophic factor or S100β may also cause the formation of basal processes in enteroendocrine cells and, as discussed below, the results presented here represent a platform to understand further the mechanisms that regulate the function of enteroendocrine cells and their neuropods.

## Discussion

The biology of enteroendocrine cells is rapidly evolving thanks to the development of transgenic mouse models in which specific hormone promoters drive the expression of green fluorescence protein [9], [10], [43]. We recently developed a Pyy-GFP mouse and identified in enteroendocrine cells of the small intestine and colon a prominent cytoplasmic process that we referred to here as a neuropod. We reasoned that its function could be derived by documenting the complete ultrastructure of the enteroendocrine cell.

Previously, documenting the ultrastructure of cells and tissues in three dimensions was limited to serial sectioning electron microscopy. An example is the seminal publication on the nervous system of a worm that stemmed from the pioneering work of Brenner and colleagues [44], [45]. Although the resolution of this method is still unmatched, its applicability has been hindered by the large amount of manual labor required to cut, image and align serial sections. To overcome this issue, Denk and Horstmann [15] developed a technology in which an ultramicrotome slices layers as thin as 20 nm from a block of tissue, and the face of the block is imaged using scanning electron microscopy. Known as SBEM, for serial-block face scanning electron microscopy, this technology is helping to unveil the ultrastructure of rare cell types, elusive synaptic connections, and distant brain neurocircuits. Here, we used SBEM along with confocal microscopy to identify a specific enteroendocrine cell in the distal small intestine and unveil its ultrastructure in the third dimension. A series of 700 micrographs were produced, spaced every 75 nm at a resolution of 5 nm/pixel, and used to segment manually the areas of interest. The results revealed a distinctive anatomy of enteroendocrine cells with features previously attributed only to neurons.

The neuropod in enteroendocrine cells has axon-like characteristics and appears to guide the secretion of hormones. We found that within the neuropod of Pyy-GFP positive cells are contained neurofilament light and medium. These types of intermediate filaments have been demonstrated in brush cells of the stomach epithelia, a type of sensory epithelial cell [46], but their function in sensory epithelial cells remains to be defined. In neurons, neurofilaments are necessary to determine the caliber of axons. Mice that carry a null mutation in neurofilament medium in the central and peripheral nervous systems have reduced neurofilament light and the diameter of axons is diminished [47]. Because the diameter of axons is proportional to the conduction velocity of electrical impulses [48], it is possible that neurofilaments in enteroendocrine cells may also influence the diameter of neuropods and the propagation of action potentials. Recently, it was demonstrated that electrical activity triggered by apical activation of calcium currents drives the secretion of hormones located in the base of enteroendocrine cells of the distal small intestine and colon [49]. Our results show that 73.5% of all secretory vesicles are distributed within the neuropod and 30% are accumulated at its tip, thus, it is likely that the axon-like process in enteroendocrine cells guides hormone secretion.

As exposed by SBEM data, the enteroendocrine cell’s neuropod is also escorted by enteric glia. This appears to be a conserved relationship because about 70% of enteroendocrine cells were in contact with a process from GFAP-positive enteric glia. Although enteric glia were first identified by neuroanatomist A. S Dogiel in 1899 [50], their essential role in intestinal function was only demonstrated in 1998 [32]. Targeted-ablation of GFAP-positive enteric glia in the small intestine causes a fulminant and fatal inflammation of the bowel. Since then, enteric glia have been shown to modulate intestinal epithelial proliferation [51], determine enteric neuron phenotypes [30], and give rise to enteric neurons in response to injury [31]. Likewise, it is possible that enteric glia modulate enteroendocrine cell function in a similar fashion. This remarkable relationship could only be seen by analyzing the ultrastructure in three dimensions because of the minute (<100 nm) spatial dimension of the cell-to-cell contact. Encouraged by this structural finding, we discovered using FAC sorting and qRT-PCR that enteroendocrine cells express several molecular receptors for neurotrophins, including NGF-β and artemin. Thus, as a proof of principle, we extended these structural findings into a novel *in vitro* intestinal organoid system that allows for the study of function [52].

Studying enteroendocrine cells *in vitro* has been challenging because, when isolated from epithelial cells, enteroendocrine cells undergo anoikis and subsequent cell death. Recently, however, the discovery of the factors that allow a single intestinal stem cell to give rise to all cell types of the intestinal epithelium, including enteroendocrine cells, has revolutionized intestinal stem cell biology [53]. This finding allows for the isolation of the intestinal compartments of stem cells – the intestinal crypt – which can then be turned into a small intestinal organoid or mini-gut [52]. Mini-guts have the main types of intestinal epithelial cells, including enteroendocrine cells. In this system, we found that the percentage of enteroendocrine cells with neuropods and the length of these neuropods was enhanced by the neurotrophic factors NGF-β and artemin. Although very few reports have addressed the function of neurotrophic factors on intestinal epithelial cells, the absence of glial derived neurotrophic factor (GDNF) alpha 2 in a knockout mouse model causes about a 30% reduction of intestinal epithelial cells that immunoreact with a synaptophysin antibody, which is a marker of enteroendocrine cells [54]. Pancreatic beta cells – another type of endocrine cell of the gastrointestinal tract – also express GDNF receptors, including GFRalpha1 and c-Ret. And, the mass and proliferation of beta cells, and the insulin they secrete is enhanced in a transgenic mouse that overexpresses GDNF in enteric glia [55]. Likewise, it is possible that enteric glia, through the secretion of neurotrophic factors, modulates the proliferation and hormone content in enteroendocrine cells. This is an important hypothesis because hormone secretion from enteroendocrine cells is altered by high-fat diets that induce obesity and intestinal inflammation [56], and enteric glia are active mediators of inflammatory responses [57], [58].

**Figure 5:**
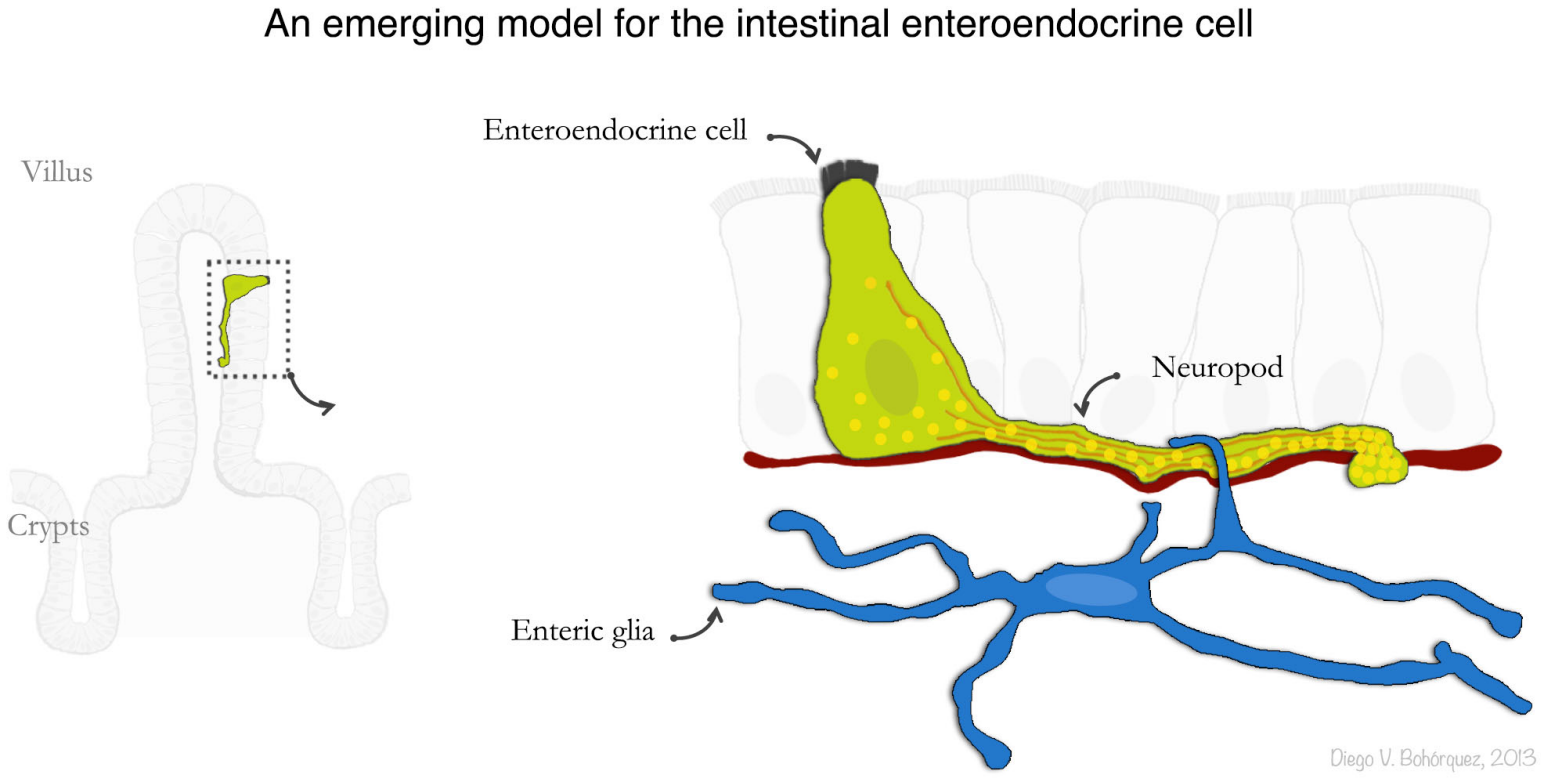
An emerging model for the enteroendocrine cell. Here we used correlative confocal-3D electron microscopy to study the ultrastructure of a single chemosensory cell in the gut. The 3D ultrastructure uncovered unique features of an axon-like neuropod in these cells, including neurofilaments, secretory vesicles, and their relationship to glia. This technical advance can be applied to similar systems in which cells of interest are rare, dispersed, and with convoluted morphology.

We therefore suggest a model in which hormone secretion in enteroendocrine cells is guided by a neuropod nurtured, in part, by glia and neurotrophic factors (Figure 5). This model originated from a technical advance that uses a cell-specific transgenic GFP mouse model in the presence of confocal microscopy and electron microscopy in three-dimensions. This is a model that can be adapted to study other elusive cell types where function can be derived from ultrastructure.

## Methods

### Mice

Animal care and experiments were carried out in accordance with protocols (registry #A050-13-02 and #A075-12-03) approved by the Institutional Animal Care and Use Committee of Duke University Medical Center. All mice were bred in house at the Division of Laboratory Animal Resources of Duke University School of Medicine. The Pyy-GFP transgenic mouse line was developed in our laboratory on a C57BL/6 background, and the methods have been previously described in Bohórquez *et al.*, 2011 [7]. The Cck-GFP transgenic line was acquired and kept on a Swiss Webster background from the Mutant Mouse Regional Resource Centers (University of Missouri, Columbia, MO).

### Fluorescence-activated Cell Sorting (FACS) and Quantitative Real Time RT-PCR (qRT-PCR)

#### Isolating cells for FACS

Intestinal and colonic epithelial cells were isolated from Pyy-GFP mice according to the method described by Sato and Clevers, 2013 [59] and fluorescence activated cell (FAC) sorted according to our established protocol [8]. *qRT-PCR.* Equal numbers of Pyy-GFP and non-GFP epithelial cells were collected in RNAprotect Cell Reagent (Qiagen, Valencia, CA) and processed following our protocol [8] for the isolation and quality control of total RNA, reverse transcription, pre-amplification, and qRT-PCR. For positive controls, we used dissociated sensory neurons from the trigeminal ganglia, which were isolated according to a published protocol [60]. The TaqMan gene expression assays (Life Technologies Corp., Carlsbad, CA) used in qRT-PCR can be found in Table S1.

#### Analysis of gene expression

Relative quantitation of gene expression was calculated using the comparative CT (ΔΔCT) method [61]. Within each sample, the GAPDH CT value was subtracted from the CT value of the target gene to calculate the ΔCT. This value was used to perform a two-tailed t-test analysis to determine statistical differences (α = 0.05) in the expression of target genes between Pyy-GFP and non-GFP epithelial cells. The value reported in graphs is the fold expression difference between Pyy-GFP and non-GFP epithelial cells and was calculated as 2^– ((ΔCTPyy − GFP) − (ΔCTnon − GFP))^.

### Intestinal Organoids

#### Crypt isolation and organoid cultures

Organoids were prepared according to the Sato and Clevers procedure [53], [59]. For each preparation, a single adult Cck-GFP mouse was used. The mouse was anesthetized with isoflurane and a ∼10 cm portion of the proximal small intestine was used to isolate the intestinal crypts. Crypts were suspended in a 1∶1 solution of basic culture medium and growth factor reduced BD Matrigel (BD, Franklin Lakes, NJ). The basic culture medium was composed of the following: advanced DMEM/F-12 (GIBCO), 200 mM L-glutamine, 100 U/mL Penicillin/Streptomycin, 10 mM Hepes, 1X N-2 (GIBCO), 1X B27 - No Vit A (GIBCO), 500 ng/mL R-Spondin1 (R&D Systems Inc., Minneapolis, MN), 50 ng/mL EGF (R&D Systems Inc., Minneapolis, MN), and 100 ng/mL Noggin (PeproTech, Rocky Hill, NJ). Crypts were then plated onto glass bottomed culture dishes (MatTek Corp., Ashland, MA) at a density of 500 crypts per plate. The gel was allowed to polymerize for 45 minutes at 37°C then overlaid with 300 µl of medium. Plates were incubated at 37°C, and medium was replaced every two days.

#### Image acquisition and analysis

After 4 days in culture, organoids were subjected to the following treatments: control (no neurotrophins), recombinant mouse beta-nerve growth factor (10, 100, or 1,000 ng/mL) (R&D Systems Inc., Minneapolis, MN), or artemin (10, 100, or 1,000 ng/mL) (R&D Systems Inc., Minneapolis, MN). All treatments were applied for 24 hours. Before imaging, culture medium was replaced with a HBSS with calcium and magnesium (GIBCO) solution containing 10 mM HEPES and 17.5 mM glucose. Each sample was examined for no more than 30 minutes at room temperature. In pilot experiments, no visible damage was observed under these conditions. Imaging was performed using a Zeiss 510 upright confocal microscope equipped with a 63X/1.0-dipping objective. At this magnification, about 30 different organoids were observed and at least 180 GFP positive cells analyzed to record the number of basal processes per cell. A basal process is defined here as any projection extending from the base of the cell. In addition, z-stacks were acquired from at least 10 GFP cells under each condition to determine the length of basal processes. These images served to measure the length of basal processes.

#### Statistical analysis

Each treatment had three biological replicates (individual mice). Each biological replicate represented the average of three dishes with organoids. The length of neuropods was measured using Fiji software. The number of cells with neuropods was expressed as a percentage of all of the cells counted. This value was transformed using the arcsin function for normal distribution. Statistical differences between treatments and control were determined using a two-tailed T-test analysis at α = 0.05.

### Immunohistochemistry

Adult mice (8–12 weeks of age) were perfused intracardially with 4% paraformaldehyde and tissues from the ileum and colon were collected for immunohistochemistry. A detailed protocol of the perfusion, tissue collection, and immunohistochemistry procedure can be found in Bohórquez *et al.*, 2011 [7]. The names, source, and concentration of primary antibodies used are listed on Table S2. Signal detection was carried out using fluorophore-conjugated secondary antibodies specific to the primary antibody species (Jackson ImmunoResearch Laboratories Inc., West Grove, PA).

### Laser-scanning Confocal Microscopy

Samples were imaged on a Zeiss 710 or 780 inverted confocal microscope using 20X/0.8 (Zeiss Plan Apochromat), 40X/1.3 (Zeiss Plan NeoFluar), or 100X/1.4oil (Zeiss Plan Apochromat) objectives. Single optical sections or z-stacks were obtained using sequential multi-track acquisition with excitation at 405 nm (DAPI), 488 nm (endogenous GFP or DyLight 488), 561 nm (Cy3), and 633 nm (DyLight 649), and emission filters of BP420–480, BP505–550, LP575, and LP650. Pinholes were set to 1 airy unit and line averaging of 4 at 1024 or 2048 pixel resolution. Differential interference contrast (DIC) images were also collected to determine the location of the cells relative to the lumen.

### Serial Block Face Scanning Electron Microscopy (Serial Block Face SEM)

#### Collecting tissue

Tissue was collected according to a published protocol [62] with the following minor modifications. Adult Pyy-GFP mice (8–12 weeks of age) were perfused intracardially for ∼1 minute with cold PBS (0.01 M, 0.9% NaCl), followed by a mixture of 4% paraformaldehyde and 0.1% glutaraldehyde for 15 minutes. While perfusion of the fixative is essential to preserve ultrastructure, reducing the concentration of glutaraldehyde is necessary to prevent autofluorescence. A piece of tissue (∼3 cm) from the distal ileum was excised and placed in a petri dish with cold PBS. The tissue segment was cut open along the mesentery and further dissected into small (∼3 mm×3 mm) blocks. Tissue blocks were then incubated in same fixative for 3 hours at 4°C.

#### Trimming tissue sections

Blocks were briefly rinsed in PBS and embedded in 5% low melting agarose previously warmed at 55°C. Low-melting agarose is preferred over conventional agarose to avoid tissue damage at temperatures higher than 55°C. Once the agarose had solidified, blocks were mounted and trimmed using a Leica VT1000 S vibrating blade microtome (Leica Microsystems Inc., Buffalo Grove, IL). Blocks were first trimmed to obtain 300 µm thick sections. These sections were then re-embedded in low melting agarose and trimmed again at 45 µm so that the final dimensions of the block were 300 µm×45 µm. In pilot experiments, we determined that a length no longer than 300 µm spans about three villi which facilitates the correlation of confocal microscopy with SBEM; whereas, a thickness of at least 45 µm is necessary to preserve the enteroendocrine cell and its entire basal process, while keeping the number of SBEM sections to a minimum.

#### Imaging tissue sections with confocal microscope

Tissue segments were stained with DAPI nuclear stain diluted in PBS (1∶5000), which served to differentiate epithelium versus lamina propria. Sections were then mounted on a glass slide using a drop of PBS and covered with a coverslip. Sections were observed under fluorescent light to identify those that had intact villi with Pyy-GFP cells of interest. Those sections that met these criteria were imaged using a Zeiss 780 inverted confocal microscope to obtain image z-stacks. In order to facilitate correlation with serial block face images, the optical sectioning interval was 1 µm.

#### Post-fixing tissue sections and embedding them in agarose

After imaging, sections were carefully removed from the glass slide using a fine art paintbrush #1 and post-fixed overnight in fresh fixative made of 4% paraformaldehyde plus 2.5% glutaraldehyde. Sections were then embedded in a thin layer of 5% low melt agarose by placing them between two glass slides. At this point, the resulting sections are almost invisible to the naked eye. Thus, embedding them flat in a thin layer of agarose eases subsequent handling. The agarose embedded sections were stored in PBS at 4°C until further processing.

#### Staining tissue sections

Samples were rinsed three times for 5 minutes each in 0.1 M cacodylate buffer and post-fixed in 0.1% tannic acid for 1 hour. This concentration of tannic acid was needed to enhance the contrast of cell membranes [63]. Thereafter, samples were stained according to the protocol of the National Center for Microscopy and Imaging Research, University of California, San Diego, CA [64].

#### Infiltrating and embedding tissue sections in resin

The tissue sections were sequentially infiltrated with resin using the EMbed 812 kit (Electron Microscopy Sciences, Hatfield, PA). The resin was prepared by mixing 24 mL of Epon 812 (Epoxy), 10.5 mL DDSA, 15.5 mL NMA, and 1 mL of DMP30. Microscope slides were used to embed the tissue sections as flat as possible. To prevent the slides from sticking to each other, slides were first cured with liquid releasing agent (Electron Microscopy Sciences, Hatfield, PA). The clear section of the slide was dipped into the liquid releasing agent, dried for 2 hours in a dust-free area, and then further dried overnight at 60°C. These slides were used to sandwich the resin-infiltrated sections. The slides with the sandwiched sections were then cured for an additional 48 hours. At this point, tissue blocks were released by pulling apart the slides.

#### Mounting the tissue block for imaging

Under a dissecting microscope, the orientation of the tissue blocks was matched with that of the confocal micrographs. Once the regions containing the cells of interest had been identified, the tissue block was trimmed manually to a block-face of approximately 500 µm×500 µm. The block was laid flat, mounted on a pin containing CircuitWorks conductive epoxy (ITW Chemtronics, Kennesaw, GA), and dried for 30 minutes. Subsequently, the block was allowed to dry on a flat surface overnight at 60°C and coated with colloidal silver liquid (Electron Microscopy Sciences, Hatfield, PA). Maintaining the tissue sections flat during embedding and trimming was imperative to allow slicing the block at a right angle. This is essential to correlate the serial block face and confocal micrographs.

#### Imaging tissue block with serial block face SEM and correlating images to confocal microscopy data

The tissue block was imaged using a Sigma VP Scanning Electron Microscope (Carl Zeiss Microscopy GmbH, Jena, Germany) equipped with a Gatan 3view system (Gatan Inc., Pleasanton, CA). The microtome was set to cut 2–5 µm increments until enough tissue was visible. Low magnification images of the entire block face were acquired at this point. Then features in both the serial block face micrographs and the confocal data were measured using Fiji software [65]. Measurements were used to generate a common denominator and account for sample morphing during staining. The common denominator was multiplied by the coordinates in the confocal data set to locate the area to image on the block face of the sample. We also took into account key structural features such as position of microvilli, goblet cells, or lamina propria to facilitate correlation. Serial block face images were acquired in high vacuum mode at a 2.25 kV and 5 nm/pixel (or 15,147X magnification) and 75 nm slices. The resulting serial block face SEM data set contained 700 images in 16-bit.dm3 format.

#### Optimizing serial block face SEM images for surface segmentation

Images were converted to 8-bit.tiff format and filtered using a 0.8 gaussian blur filter in Fiji. To minimize the amount of computer RAM memory needed to handle the data set, images were scaled down to 25% of their original size, the z dimension scaled 1∶2, and the set saved as.tiff stack. The stack was then aligned using the Fiji plugin “linear stack alignment with SIFT” in translation mode and cropped using the “crop 3D” plugin to focus on the region containing the cell of interest.

### Surface Segmentation and 3D Data Visualization

Segmentation for both confocal and serial block face SEM data was performed using Imaris 7.5 (Bitplane, South Windsor, CT).

#### Confocal microscopy

Surface segmentation of image z-stacks was performed in surpass mode using the software’s “surfaces” tool and in automatic creation mode. Each channel was reconstructed separately with the smooth option on, the surfaces area detail at 0.126 µm, and thresholding was calculated as absolute intensity.

#### Serial block face SEM

Surface segmentation of images was performed manually using the “surfaces” tool in draw mode. Areas of interest (*e.g.* cell nuclei, cell membranes) were defined using the “contour” option in drawing mode time at 50 milliseconds. Contours were drawn for every slice where the region of interest was visible. To create the surface, the resolution was kept in auto mode. Secretory vesicles were identified using the “spots” tool with the point style option in sphere mode and the radius adjusted to match the size of the secretory vesicles. Vesicles were traced in the following three groups: 1 µm above the nucleus until apical end, around the nucleus, and neuropod. The number of vesicles was determined using the spots statistics option. For both, confocal and serial block-face data, images (6000×6000 pixels) were produced using the “snapshot” tool at 300 dpi resolution. Likewise, videos were produced using the “animation” tool by adding frames manually and the final recording done at 24 frames per second. Titles and captions in the videos were added using Adobe Premier Pro CC (Adobe Systems Inc., San Jose, CA). When necessary, the size of the image was adjusted using Photoshop CS5 (Adobe Systems Inc., San Jose, CA) to fit into figure panels.

## Supporting Information

Figure S1 **Correlative confocal microscopy-SBEM method.** This additional example of confocal microscopy and SBEM correlation in the colon highlights the fidelity of the method to obtain the ultrastructure of a specific cell in 3D. Blue = Dapi nuclear stain; Green = Pyy-GFP. Bar = 1 µm.
(TIF)

Figure S2 **Resolution of SBEM data.** A 5 nm/pixel resolution allows clear identification of microvilli, plasma membranes, and secretory vesicles. Bar = 1 µm.
(TIF)

Figure S3 **Characteristics of enteric glia.** Enteric glia (blue) were identified by the following characteristics. (Left) Elongated nuclei with radial processes emanating from the body of the cell. (Middle) These processes extend to and wrap around a nerve (red). Although in the image the nerve in red appears as two pieces, this nerve is one continuous track. (Right) Glia envelope nerve tracks made of several fibers; this nerve track is composed of about 12 individual fibers. Bars = 1 µm.
(TIF)

Figure S4 **SBEM data showing enteric glia contacting enteroendocrine-like cell.** A putative enteroendocrine cell is contacted by glia-like process. Small clear vesicles can be observed in the upper tip of glial process. Bar = 1 µm.
(TIF)

Figure S5 **Confocal microscopy data showing GFAP enteric glia contacting Pyy-GFP enteroendocrine cell.** Glial fibrillary acidic protein (GFAP) immunoreactive enteric glia contacts Pyy-GFP enteroendocrine cell. Inset on the left shows, enteroendocrine cell position within the villus. Notice that there is only one GFAP positive enteric glia in the villus. On the right can be seen that individual processes from enteric glia contact each of the Pyy-GFP neuropods. Arrowheads indicate in the longer Pyy-GFP neuropod the existence of filipodia-like structures similar to those found in axonal growth cones. This is a maximal-intensity projection of Pyy-GFP ileum tissue stained with a rabbit antibody against mouse GFAP. Bar = 10 µm.
(TIF)

Table S1 **Taqman probes used in quantitative RT-PCR.**
(PDF)

Table S2 **Primary antibodies used for immunofluorescence.**
(PDF)

Video S1 **The ultrastructure of the enteroendocrine cell in 3D.** Volume segmentation was performed on the SBEM data set to reveal the ultrastructure of all epithelial nuclei and an enteroendocrine cell. Mitochondria, secretory vesicles, and neurofilaments were found accumulated within the enteroendocrine cell neuropod.
(MOV)

Video S2 **Neuropod of Pyy-GFP enteroendocrine cell contains neurofilament medium.** This is a confocal z-stack 15 µm thick from the ileum of a Pyy-GFP mouse. Immunohistochemistry was performed to reveal neurofilament medium. An antibody against GFP was used to preserve fluorescence during immunohistochemistry.
(MOV)

Video S3 **Enteroendocrine cells and enteria glia connect.** Volume segmentation was performed on SBEM data to reveal an enteroendocrine cell and enteric glia connection.
(MOV)
