# Supplementary figures and images for: An Enteroendocrine Cell – Enteric Glia Connection Revealed by 3D Electron Microscopy
Source: PLoS One. 2014 Feb 26;9(2):e89881. doi: 10.1371/journal.pone.0089881 (PMC3935946; doi:10.1371/journal.pone.0089881)

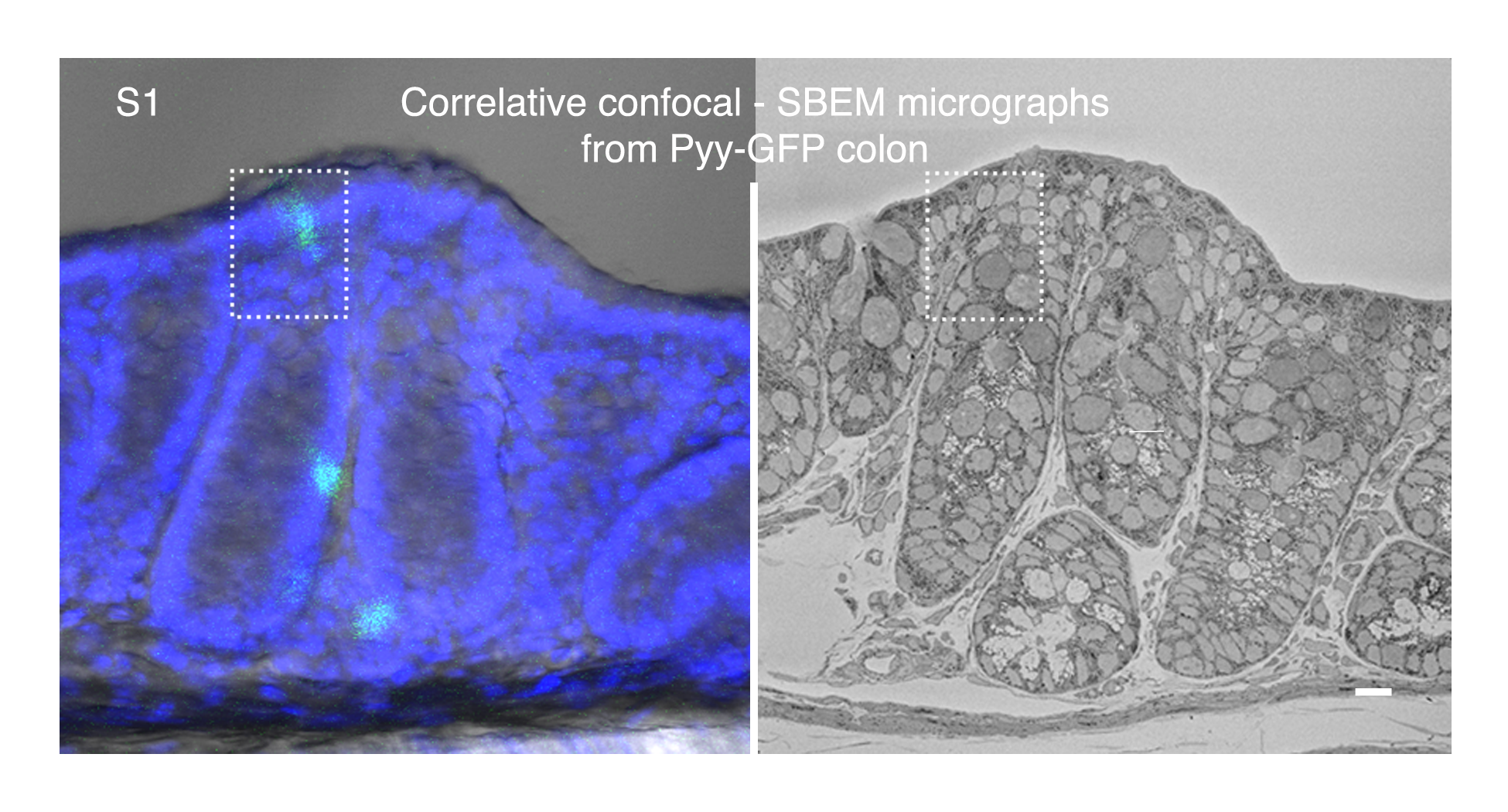

Supplement: Figure S1 — Correlative confocal microscopy-SBEM method. This additional example of confocal microscopy and SBEM correlation in the colon highlights the fidelity of the method to obtain the ultrastructure of a specific cell in 3D. Blue = Dapi nuclear stain; Green = Pyy-GFP. Bar = 1 µm. (TIF) [file pone.0089881.s001.tif]

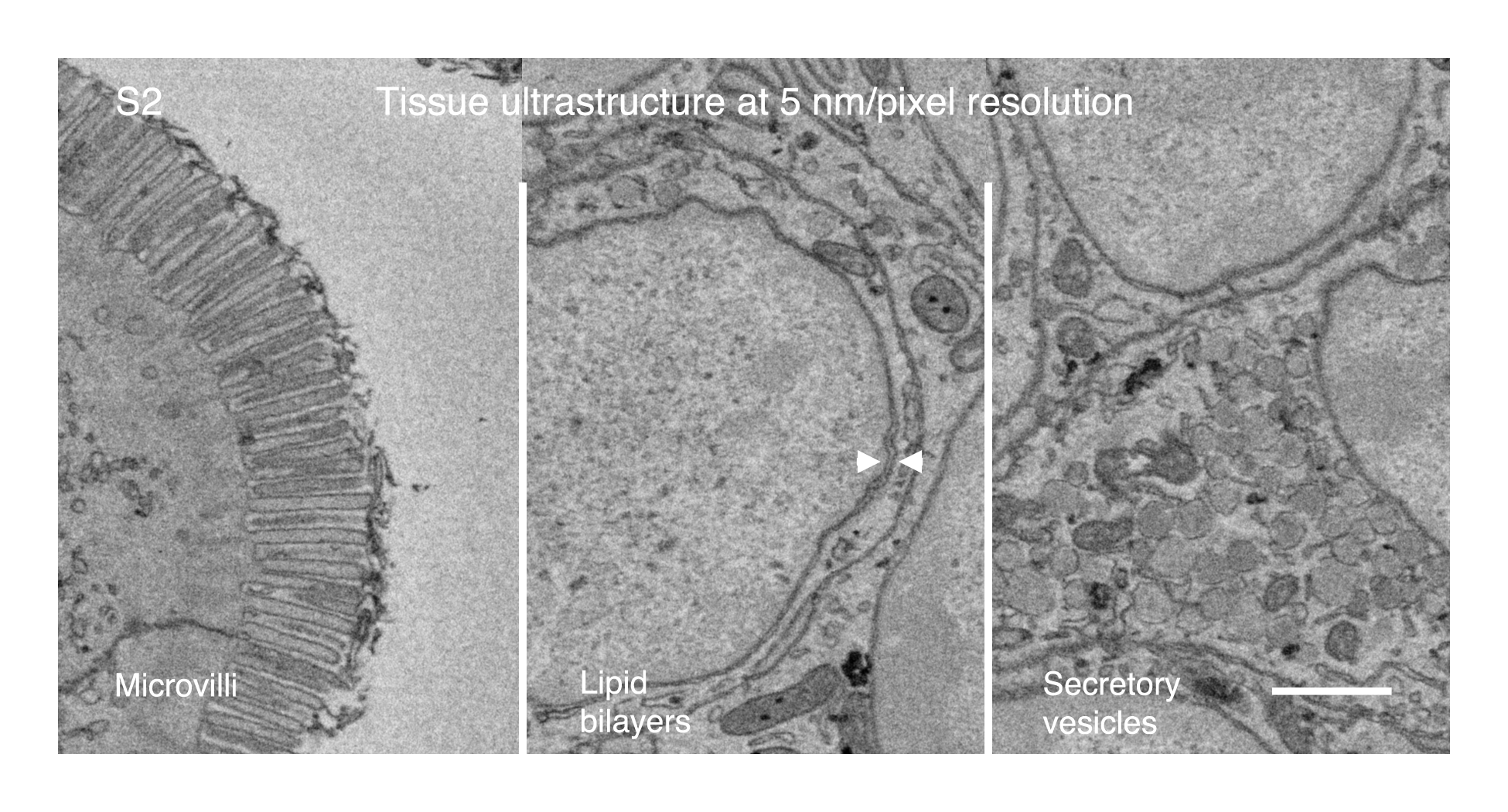

Supplement: Figure S2 — Resolution of SBEM data. A 5 nm/pixel resolution allows clear identification of microvilli, plasma membranes, and secretory vesicles. Bar = 1 µm. (TIF) [file pone.0089881.s002.tif]

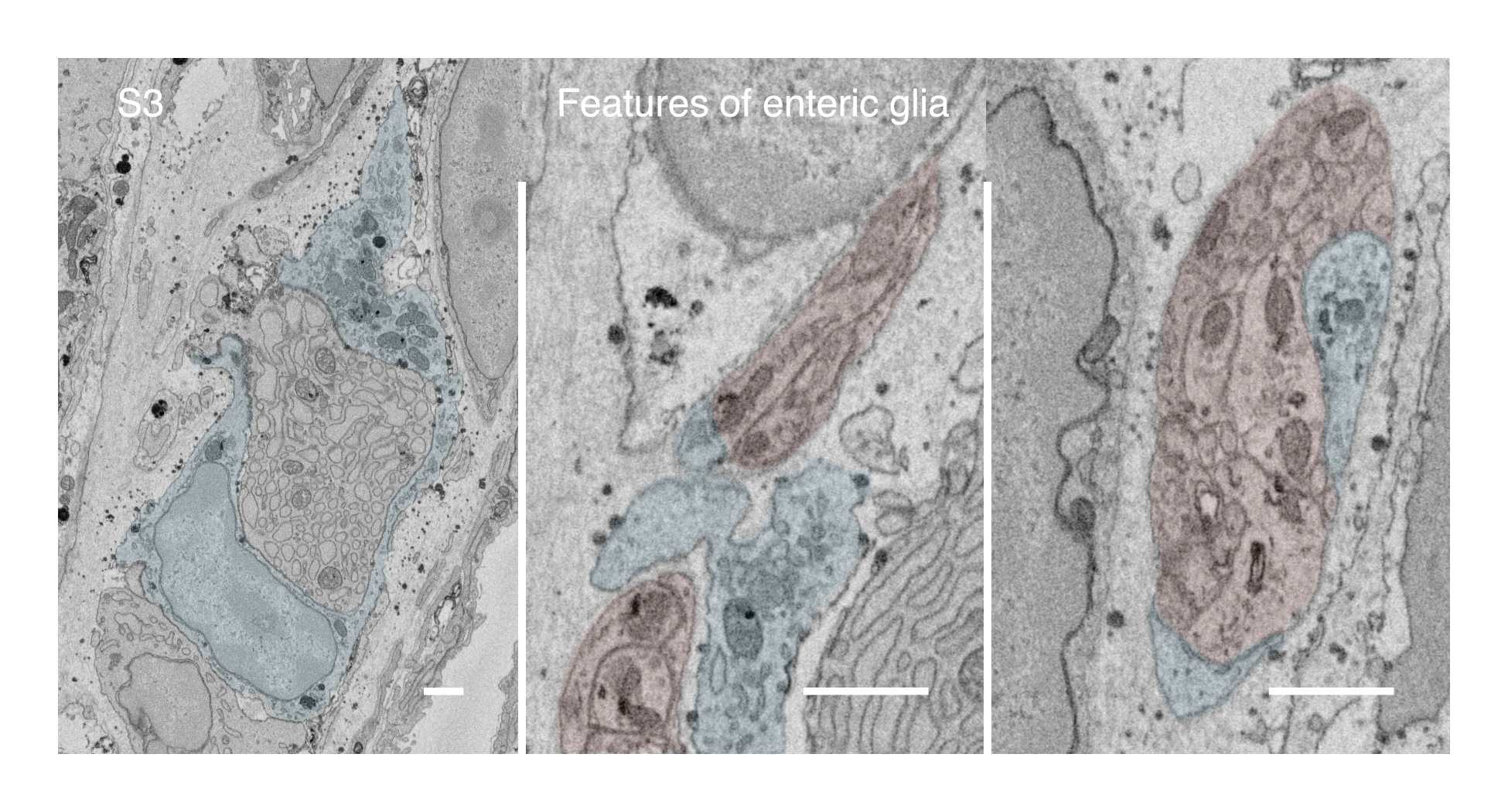

Supplement: Figure S3 — Characteristics of enteric glia. Enteric glia (blue) were identified by the following characteristics. (Left) Elongated nuclei with radial processes emanating from the body of the cell. (Middle) These processes extend to and wrap around a nerve (red). Although in the image the nerve in red appears as two pieces, this nerve is one continuous track. (Right) Glia envelope nerve tracks made of several fibers; this nerve track is composed of about 12 individual fibers. Bars = 1 µm. (TIF) [file pone.0089881.s003.tif]

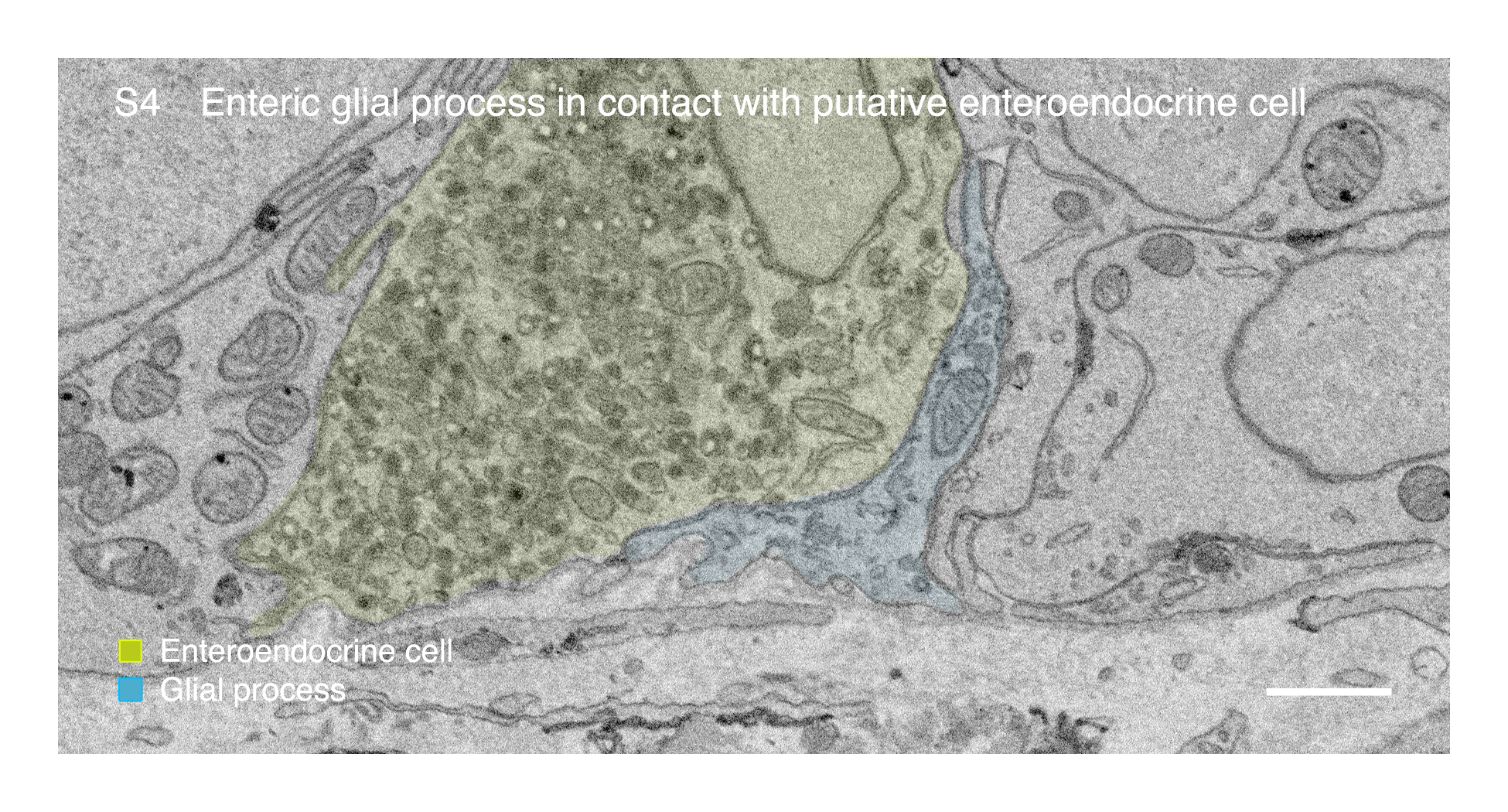

Supplement: Figure S4 — SBEM data showing enteric glia contacting enteroendocrine-like cell. A putative enteroendocrine cell is contacted by glia-like process. Small clear vesicles can be observed in the upper tip of glial process. Bar = 1 µm. (TIF) [file pone.0089881.s004.tif]

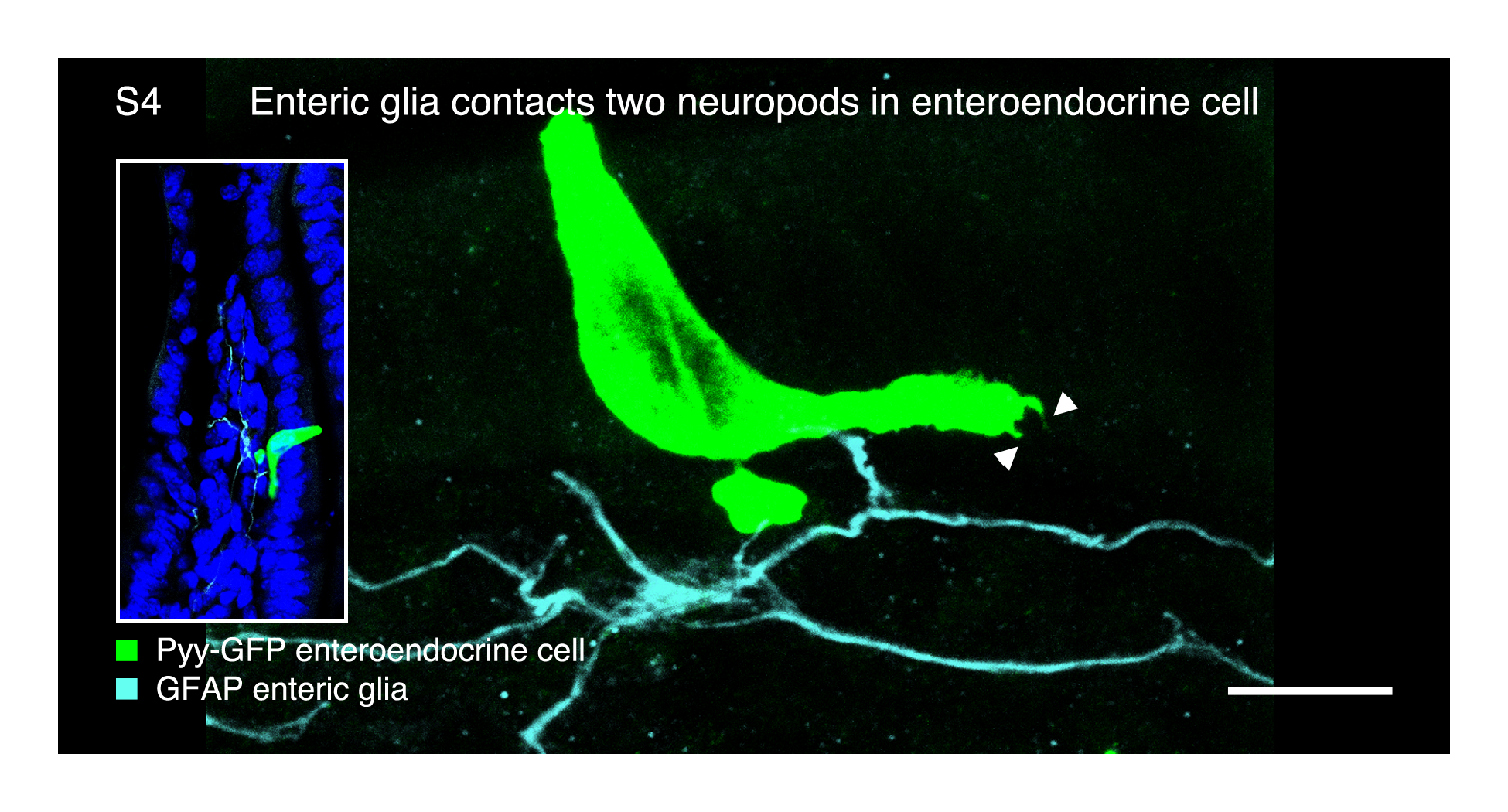

Supplement: Figure S5 — Confocal microscopy data showing GFAP enteric glia contacting Pyy-GFP enteroendocrine cell. Glial fibrillary acidic protein (GFAP) immunoreactive enteric glia contacts Pyy-GFP enteroendocrine cell. Inset on the left shows, enteroendocrine cell position within the villus. Notice that there is only one GFAP positive enteric glia in the villus. On the right can be seen that individual processes from enteric glia contact each of the Pyy-GFP neuropods. Arrowheads indicate in the longer Pyy-GFP neuropod the existence of filipodia-like structures similar to those found in axonal growth cones. This is a maximal-intensity projection of Pyy-GFP ileum tissue stained with a rabbit antibody against mouse GFAP. Bar = 10 µm. (TIF) [file pone.0089881.s005.tif]
