# Supplementary material for: An Enteroendocrine Cell – Enteric Glia Connection Revealed by 3D Electron Microscopy
Source: PLoS One. 2014 Feb 26;9(2):e89881. doi: 10.1371/journal.pone.0089881 (PMC3935946; doi:10.1371/journal.pone.0089881)
Supplement: Table S1 — Taqman probes used in quantitative RT-PCR. (PDF) [file pone.0089881.s006.pdf]

Table S1. Taqman probes used in quantitative RT-PCR.

Gene Name	Common function	Symbol	Cat. No.
Glyceraldehyde 3-phosphate dehydrogenase	House keeping gene	Gapdh	Mm99999915_g1
Peptide YY	Neuropeptide	Pyy	Mm00520716_g1
Colecystokinin	Neuropeptide	Cck	Mm00446170_m1
Neurofilament heavy	Axonal proteins	Nefh	Mm01191456_m1
Neurofilament medium	Axonal proteins	Nefm	Mm00456201_m1
Neurofilament light	Axonal proteins	Nefl	Mm01315666_m1
Glial cell derived neurotrophic factor receptor alpha 1	Axonal growth	Gfra1	Mm00833897_m1
Glial cell derived neurotrophic factor receptor alpha 3	Axonal growth	Gfra3	Mm00494589_m1
Neurotrophic tyrosine kinase, receptor, type 1	Axonal growth	TrkA	Mm01219406_m1
Neurotrophic tyrosine kinase, receptor, type 2	Axonal growth	TrkB	Mm00435422_m1
Neurotrophic tyrosine kinase, receptor, type 3	Axonal growth	TrkC	Mm00456222_m1
Nerve growth factor receptor	Axonal growth	Ngfr	Mm01309635_m1
