# Supplementary material for: An Enteroendocrine Cell – Enteric Glia Connection Revealed by 3D Electron Microscopy
Source: PLoS One. 2014 Feb 26;9(2):e89881. doi: 10.1371/journal.pone.0089881 (PMC3935946; doi:10.1371/journal.pone.0089881)
Supplement: Table S2 — Primary antibodies used for immunofluorescence. (PDF) [file pone.0089881.s007.pdf]

Table S2. Primary antibodies used for immunofluorescence.

Target	Symbol	Species	Dilution†	Source	Cat. #.
Green fluorescent protein	GFP	Chicken	1:1000	Abcam	ab13970
Green fluorescent protein	GFP	Goat	1:1000	Abcam	ab6673
Peptide YY (aa 4-21)[1]	PYY	Rabbit	1:2000	Biosource	Custom made
Cholecystokinin	CCK	Rabbit	1:2000	Biosource	Custom made
Glial fibrillary acidic protein	GFAP	Rabbit	1:2000	Dako	Z0334
S100-β	S100-β	Rabbit	1:500	Abcam	ab41548
α–smooth muscle actin	α-SMA	Rabbit	1:500	Abcam	ab5694
Neurofilament light	NFl	Rabbit	1:1000	Abcam	ab9035
Neurofilament medium	NFm	Rabbit	1:2000	Abcam	ab9034
Neurofilament medium	NFm	Mouse	1:500	Abcam	ab7794
Neurofilament heavy	NFh	Rabbit	1:2000	Abcam	ab8135
Neurofilament heavy	NFh	Chicken	1:500	Abcam	ab72996

†All antibodies were diluted in 50mM TRIS-buffered saline containing 0.05% Tween-20 and 0.1% bovine serum albumen.
